# Comparative transcriptome sequencing analysis of female and male *Decapterus macrosoma*

**DOI:** 10.7717/peerj.14342

**Published:** 2022-11-08

**Authors:** Zizi Cai, Shigang Liu, Wei Wang, Rui Wang, Xing Miao, Puqing Song, Binbin Shan, Liangming Wang, Yuan Li, Longshan Lin

**Affiliations:** 1 Third Institute of Oceanography, Ministry of Natural Resources, Xiamen, China; 2 Key Laboratory of Marine Ranching, Ministry of Agriculture and Rural Affairs, Guangzhou, China; 3 Key Laboratory of Marine Ecological Conservation and Restoration, Ministry of Natural Resources, Xiamen, China

**Keywords:** *De novo* assembly, *Decapterus macrosoma*, Transcriptome, Growth-related genes, Sex-related genes, Differentially expressed genes

## Abstract

Sexual growth dimorphism is a common phenomenon in teleost fish and has led to many reproductive strategies. Growth- and sex-related gene research in teleost fish would broaden our understanding of the process. In this study, transcriptome sequencing of shortfin scad *Decapterus macrosoma* was performed for the first time, and a high-quality reference transcriptome was constructed. After identification and assembly, a total of 58,475 nonredundant unigenes were obtained with an N50 length of 2,266 bp, and 28,174 unigenes were successfully annotated with multiple public databases. BUSCO analysis determined a level of 92.9% completeness for the assembled transcriptome. Gene expression analysis revealed 2,345 differentially expressed genes (DEGs) in the female and male *D. macrosoma*, 1,150 of which were female-biased DEGs, and 1,195 unigenes were male-biased DEGs. Gene Ontology (GO) and Kyoto Encyclopedia of Genes and Genomes (KEGG) analyses showed that the DEGs were mainly involved in biological processes including protein synthesis, growth, rhythmic processes, immune defense, and vitellogenesis. Then, we identified many growth- and sex-related genes, including *Igf*, *Fabps*, EF-hand family genes, *Zp3*, *Zp4* and *Vg*. In addition, a total of 19,573 simple sequence repeats (SSRs) were screened and identified from the transcriptome sequences. The results of this study can provide valuable information on growth- and sex-related genes and facilitate further exploration of the molecular mechanism of sexual growth dimorphism.

## Introduction

Sexual growth dimorphism, which refers to growth rate differing between the sexes, is a common phenomenon in teleost fish. In certain fish species, such as tilapia, males grow faster and larger than females (Toguyeni et al., 1997), while in chinook salmon (*Oncorhynchus tshawytscha*), common carp (*Cyprinus carpio*), and half-smooth tongue sole (*Cynoglossus semilaevis*), females grow significantly faster and larger than males (Ma et al., 2011). And the mechanism of sexual growth dimorphism is complex (Rennie et al., 2008).

Fish growth is a complex polygenic trait that is regulated by many factors, including nutrition, environment, reproductive activity, and energy metabolism (Wang et al., 2018). Fortunately, sexually dimorphic growth can be exploited to explore candidate networks and genes that enhance body size or growth speed, which may lead to rapid and significant economic gains (Dutney, Elizur & Lee, 2017). Based on analyses of genes that are differentially expressed between females and males, an increasing number of growth-related genes have been identified (Wang et al., 2018; Lou et al., 2018; Ma et al., 2016). Sex is one of the most intriguing characteristics in the life sciences. Associated mechanisms are highly conserved in many animals (*e.g*., mammals and birds) (Yang et al., 2015; Chen et al., 2017). Many of the genes involved in sexual differentiation are identical in distantly related species, indicating that the gene regulatory mechanisms of sex differentiation and determination may be similar (Ottolenghi et al., 2007; Smith et al., 2009). However, the sex determination mechanisms in teleost fish are quite different (Devlin & Nagahama, 2002; Ribas et al., 2016). Among animals, teleost fish exhibit the most diverse sex determination system, leading to many reproductive strategies (Kobayashi, Nagahama & Nakamura, 2013; Casas et al., 2016). Unlike sex in birds, humans, and other higher vertebrates, which is relatively stable, sex in teleost fish is unstable or variable (Barske & Capel, 2008). The study of sex-related genes in teleost fish has attracted wide interest from an increasing number of researchers. As teleost fish are important protein resources for humans, understanding the growth and sex of teleost fish is important for predicting potential impacts and for effective management.

**Figure 1:**
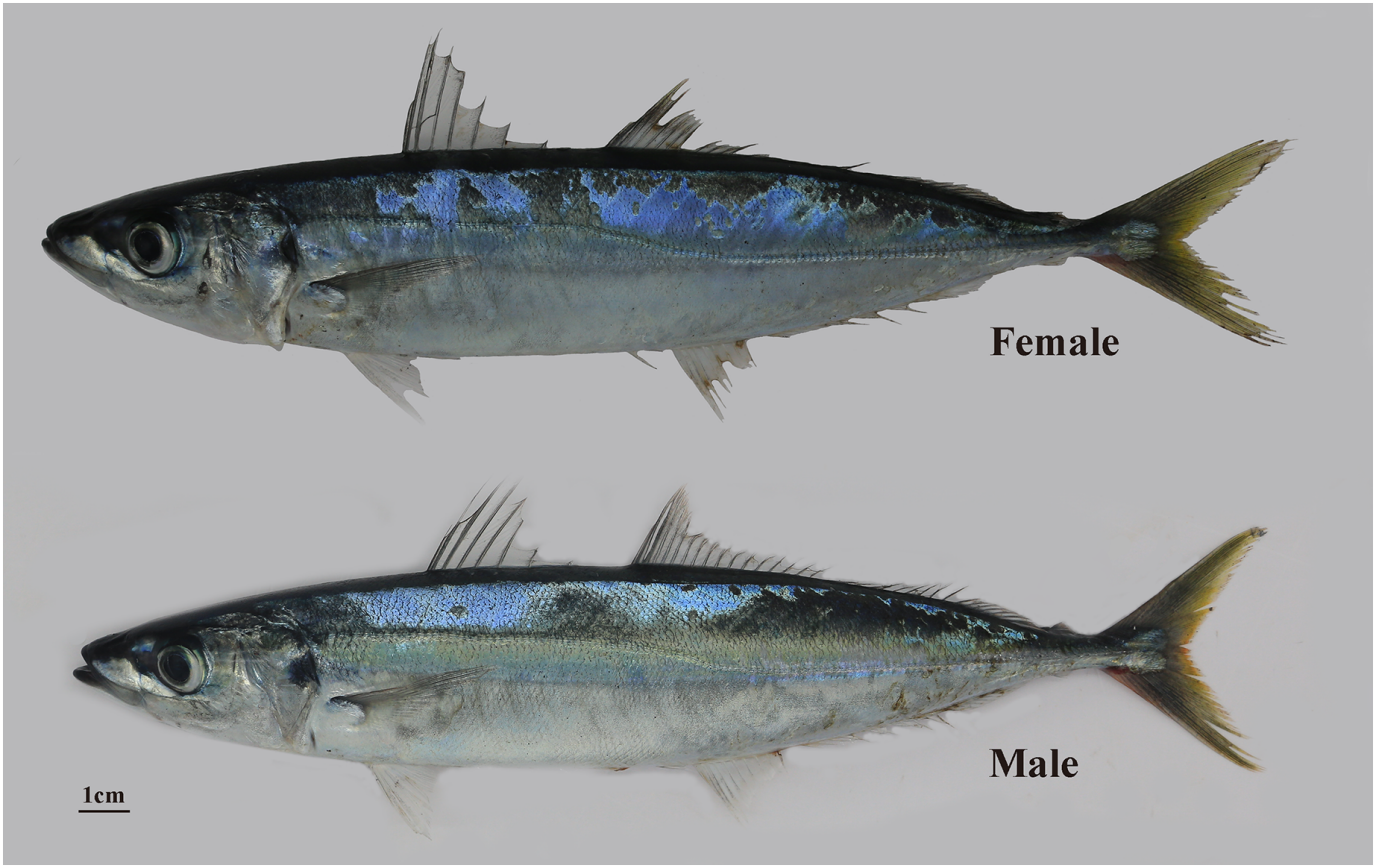
*D. macrosoma* Bleeker, 1851. The top of the picture is the female *D. macrosoma* and the bottom is the male *D. macrosoma*.

*Decapterus macrosoma* (Fig. 1) Bleeker, 1851 (Actinopterygii, Perciformes, Carangidae, *Decapterus*) usually swims in schools and generally inhabits open water at depths of 20–170 meters. It feeds on planktonic invertebrates and is mainly distributed in the East China Sea, the South China Sea, the waters around Taiwan, the south of Tokyo Bay in Japan, and the warm-water areas of the Indian-Pacific Ocean (Institute of Zoology, Chinese Academy of Sciences, Institute of Oceanology, Chinese Academy of Sciences & Shanghai Ocean University, 1962). *D. macrosoma* is widely distributed in the South China Sea, is the main target of light seining fisheries there, and has high economic value (Yang et al., 2009; Zhang et al., 2016a, 2016b, 2016c). Carangidae fish play a significant role in global marine fisheries, and annual *Decapterus* production is second only to that of *Trachurus* (Zhang et al., 2020). *D. macrosoma* exhibits sexual growth dimorphism; similar to various teleost fish; the female fish tend to be stronger and larger than the male fish (Shan et al., 2021a). These biological features render *D. macrosoma* an ideal non-model marine fish species for growth- and sex-related gene research. However, the National Center for Biotechnology Information (NCBI) database has very little genome assembly data (only 14 data points) for Carangidae fishes and no genome assembly data for *Decapterus*.

Genome sequencing in researched species can be classified based on the availability of reference genome sequences for the species, *de novo* sequencing (species without reference genomic sequences), and whole-genome resequencing (species with reference genomic sequences) (Dong, 2018). The latter is not applicable to *D. macrosoma* since it has no available genome assembly data in the public database. Thus, *de novo* sequencing was adopted in this study. Despite the lack of genomic information on *D. macrosoma*, transcriptome sequencing could reveal the relationships among genes, the biological characteristics of this species and the external environment, contributing to the future development of many effective gene resources (Sánchez et al., 2011).

To date, studies on *D. macrosoma* in China and elsewhere have mainly focused on growth and reproduction and on resource assessment (Kong et al., 2019; Hou et al., 2020); few studies on the *D. macrosoma* transcriptome have been reported. In this study, the gonads, livers, hearts, kidneys, and muscle tissues of females and males were used for transcriptome analysis. Then, gene expression profiles of different *D. macrosoma* individuals were generated, and differentially expressed genes (DEGs) between *D. macrosoma* males and females were detected. Subsequently, important candidate genes and pathways involved in growth-related and sex-related genes were identified. In addition, abundant simple sequence repeats (SSRs) were detected in the reads of deep coverage sequence regions. The aim of this study was to detect growth-related and sex-related genes in teleost fish and to lay the foundation for revealing the sexual growth dimorphism mechanisms of teleost fish. In addition, the transcriptomic resource in this study can provide support for future studies on molecular regulatory mechanisms in teleost fish.

## Materials and Methods

### Ethics approval and sample collection

All the experimental procedures were approved by the ethics committee of Laboratory Animal Welfare and Ethics of South China Sea Fisheries Research Institute (accession no. nhdf 2022-06). The procedures involving animals in this study were conducted in accordance with the Laboratory Animal Management Principles of China. The *D. macrosoma* specimens sampled in this study were captured in the South China Sea (10°N, 110°30′E) in July 2019 using light seine nets. The ovaries of female *D. macrosoma* are reddish-yellow, and the spermathecae of *D. macrosoma* are milky white and flattened in bands. Three healthy female and three healthy male fish (female fork length: 213–226 mm; male fork length: 208–218 mm) at stage IV sexual maturity were selected and adaptively raised in aerated, circularly flowing seawater for 6 d. During the adaptation period, the fish were not fed to minimize contaminating sequences from food. Then, all specimens were anaesthetized and killed by severing the spinal cord. Then, the gonads, livers, hearts, kidneys, and muscle tissues were rapidly removed, stored temporarily in liquid nitrogen, and then preserved in a −80 °C freezer for later RNA extraction. Before RNA extraction, the gonads, livers, hearts, kidneys, and muscle tissues were separately ground with liquid nitrogen, and all the tissues were blended in equal amounts to generate mixed samples.

### RNA extraction and illumina sequencing

Total RNA was extracted from the six samples using TRIzol reagent. Then, RNA integrity was assessed using an Agilent 2100 Bioanalyzer (Agilent Technologies, Palo Alto, CA, USA), and samples with an RNA Integrity Number (RIN) ≥7 were used for subsequent cDNA library preparation (Zhang, Lou & Han, 2019). The RNA of tissues from every individual was pooled in equal amounts. The statistical power of this experimental design, calculated in RNASeqPower, is 0.80 (Hart et al., 2013).

Then, mRNA (RNA with a poly-A tail) was extracted from the total RNA using magnetic beads with Oligo (dT) probes and purified. Fragmentation buffer was applied to lyse the mRNA into fragments with a suitable size, and the fragmented mRNA was reverse-transcribed into double-stranded cDNA by the N6 random primer. Then, the cDNA fragments were repaired with phosphate at the 5′ end, and an “A” base was added to the 3′ end, after which adaptors were ligated to the cDNA fragments. PCR was performed to amplify the ligation products. After thermal denaturation, the single-stranded DNA was cyclized using splint oligonucleotides and DNA ligase (Shan et al., 2021b). Finally, the cDNA libraries were sequenced on the Illumina NovaSeq high-throughput sequencing platform based on sequencing-by-synthesis (SBS) technology, thus generating many high-quality reads. The reads or bases generated by the sequencing platform were the raw data and were saved in FASTQ format, and the quality score of most bases reached or exceeded Q30. The raw data from each sequenced sample included two FASTQ files, each containing the reads determined at both ends of all cDNA fragments.

### *De novo* assembly and functional annotation

Reads containing sequencing adaptors and primer sequences were excluded from all the original sequences *via* Trimmomatic (v 0.35) (Bolger, Marc & Bjoern, 2014), and low-quality data were filtered out to ensure that we obtained high-quality clean reads (Wang, Lou & Shui, 2019). Afterward, the Trinity (v 2.11.0) software package was applied to assemble the high-quality clean reads *de novo* (Grabherr et al., 2013). First, the sequencing reads were broken into short fragments (K-mers), which were then extended into long fragments (contigs). Next, the long fragments were overlapped to generate fragment sets. Finally, transcript sequences were separately identified from each fragment set based on de Bruijn graphs and sequencing read information. Based on the identification outcomes, different contigs from the same transcript were connected using double-end information for further sequence splicing, yielding a transcript. The lead transcript in each transcript clustering unit was selected as the unigene sequence, and cluster analysis and further elimination of redundancy in the unigene data were conducted *via* the TIGR Gene Indices clustering tools (TGICL); nonredundant unigenes were ultimately obtained (Chen et al., 2016). To perform the quantitative assessment of assembly and completeness, BUSCO software version 5.4.3 (Benchmarking Universal Single-Copy Orthologs) was applied using default setting (Simão et al., 2015). Finally, to obtain information on the unigenes, comparison software, including Diamond (Buchfink, Xie & Huson, 2015) and BLAST, was used to compare all the unigenes with those in databases, including Cluster of Orthologous Groups of Proteins (COG), Gene Ontology (GO), the Kyoto Encyclopedia of Genes and Genomes (KEGG), euKaryotic Orthologous Groups (KOG), protein family (Pfam), the Swiss-Prot protein (Swiss-Prot) database, and the nonredundant protein (Nr) database.

### Gene expression and differential expression analysis

The transcriptome data obtained by Trinity splicing were used as reference sequences to estimate the gene expression level of each sample by RSEM (Dewey & Li, 2011). Next, we used the short reads alignment tool Bowtie2 to map all unigenes to the assembled transcriptome, which served as a reference (Langmead, 2009). The fragments per kilobase of exon model per million mapped fragments (FPKM) value was used to denote the expression abundance of the corresponding unigenes; this value can eliminate the influence of gene length and sequencing quality differences on the calculation of gene expression (Trapnell et al., 2010). To show the relationships among the six samples, we used the FactoMineR in R to perform a principal component analysis (PCA). To detect DEGs, the clean data were aligned with the assembled reference sequences, and the number of reads per gene was obtained based on the results. Then, the read count data were standardized using the trimmed mean of M-values (TMM), followed by differential expression analysis *via* DEGseq (Love, Huber & Anders, 2014). In this process, the well-established Benjamini-Hochberg method was used to correct the significance (Q-value) obtained from testing the original hypothesis. Moreover, the Q-value, *i.e*., the false discovery rate (FDR), was used as the key index for screening DEGs to reduce the number of false-positive results induced by the independent statistical hypothesis testing of the expression levels of many genes. The thresholds for screening were Q-value ≤ 0.01 and |log_2_ fold change| ≥ 1 (Wang et al., 2010).

### Functional enrichment analysis of DEGs

The obtained DEGs were subjected to GO enrichment analysis. Using the GO annotation results, significant enrichment analysis was conducted on the DEGs from the transcriptomes of female and male *D. macrosoma* using GOseq in R. According to the results of the GO analysis combined with biological significance, the genes for subsequent studies were selected to analyze the biological functions of DEGs (Shan et al., 2021a).

A pathway analysis was performed on the DEGs using KEGG annotations. The significance of the DEG enrichment in each pathway was calculated using the hyper geometric distribution test, and a *p*-value ≤0.05 indicated significantly enriched terms. Next, the DEG enrichment in KEGG pathways was determined *via* KOBAS (v 2.0) (Xie et al., 2011). The functional enrichment and pathway enrichment results for the DEG unigenes from *D. macrosoma* were visualized using R software (Dong et al., 2020).

### Simple sequence repeat marker detection

The MIcroSAtellite identification tool (MISA, v 2.1) was applied to identify the SSR loci in the assembled *D. macrosoma* transcript reference (Sebastian et al., 2017). The thresholds of the minimum repeat number for various unit types were set as 1-10, 2-6, 3-5, 4-5, 5-5, and 6-5. Taking the threshold 1-10 as an example, this setting indicated that a single nucleotide repeat type was repeated at least 10 times before it was considered a microsatellite.

## Results

### Sequencing and assembly of the *D. macrosoma* transcriptome

Transcriptome sequencing was performed on the six *D. macrosoma* samples using Illumina NovaSeq high-throughput sequencing. After quality control, a total of 42.61 Gb of clean data were acquired, and the Q30 base percentage in each sample was at least 93.43%. The sequencing data (clean data) from the six *D. macrosoma* samples are summarized in Table S1. Transcriptome assembly was completed after clustering by removing redundancy with Trinity and TGICL, and the detailed results for the assembled unigenes of each sample are presented in Table S2. The length distribution of unigenes in the *D. macrosoma* transcriptome are displayed in Fig. S1. The results of the BUSCO analysis showed that 92.9% were completed (237 BUSCOs), 2.7% were fragmented (seven BUSCOs), and 4.4% were missing (11 BUSCOs).

The raw reads in this study are archived in the NCBI Short Read Archive (SRA) databases under BioProject PRJNA825736, with accession numbers SRR18748694–SRR18748699. This Transcriptome Shotgun Assembly project was deposited at DDBJ/EMBL/GenBank under the accession GJWF00000000. The version described in this article is the first version, GJWF01000000.

### Functional annotation of unigenes

#### Calculation of the success rates of gene function annotation

The unigenes were further functionally characterized based on the description of their similar sequences. The number and percentage of unigenes matched to various databases are shown in Table S3. All unigenes were compared against the databases listed. The results indicated that 28,174 (48.18%) unigenes matched homologous sequences from at least one database.

#### Analysis of gene function annotation

The unigenes in the transcriptome were compared against the NCBI protein database Nr to acquire *D. macrosoma* gene sequences and their functional information and to further determine the gene sequence similarity between *D. macrosoma* and its sister species. The *D. macrosoma* transcriptome contained many unigenes that were highly similar to gene sequences from *Stegastes partitus* (7,552), *Larimichthys crocea* (6,312), *Oreochromis niloticus* (1,916), *Notothenia coriiceps* (1,108), *Maylandia zebra* (785), *Cynoglossus semilaevis* (725), *Astatotilapia burtoni* (646), *Neolamprologus brichardi* (562), *Haplochromis nyererei* (515), and *Danio rerio* (461). The other 5,932 unigenes showed similarities to gene sequences from 394 other species (Fig. S2).

A total of 23,633 unigenes were grouped into 42 functional classes across the three major GO categories (cellular component, biological process, and molecular function). Among the 23 functional classes under biological process, “cellular process” (14,321 unigenes) and “biological regulation” (9,126 unigenes) contained the most genes; among the six functional classes under cellular component, “cellular anatomical entity” (14,940 unigenes) and “intracellular” (8,916 unigenes) contained the most genes; and among the 13 functional classes under molecular function, “binding” (13,234 unigenes) and “catalytic activity” (7,120 unigenes) contained the most genes.

KEGG pathway enrichment indicated that 22,890 unigenes significantly matched to 343 pathways. Among them, “mitogen-activated protein kinase (MAPK) signaling pathway” (569) contained the most unigenes, followed by “endocytosis” (544) and “calcium signaling pathway” (502).

### DEG expression and enrichment analysis

#### DEG screening

**Figure 2:**
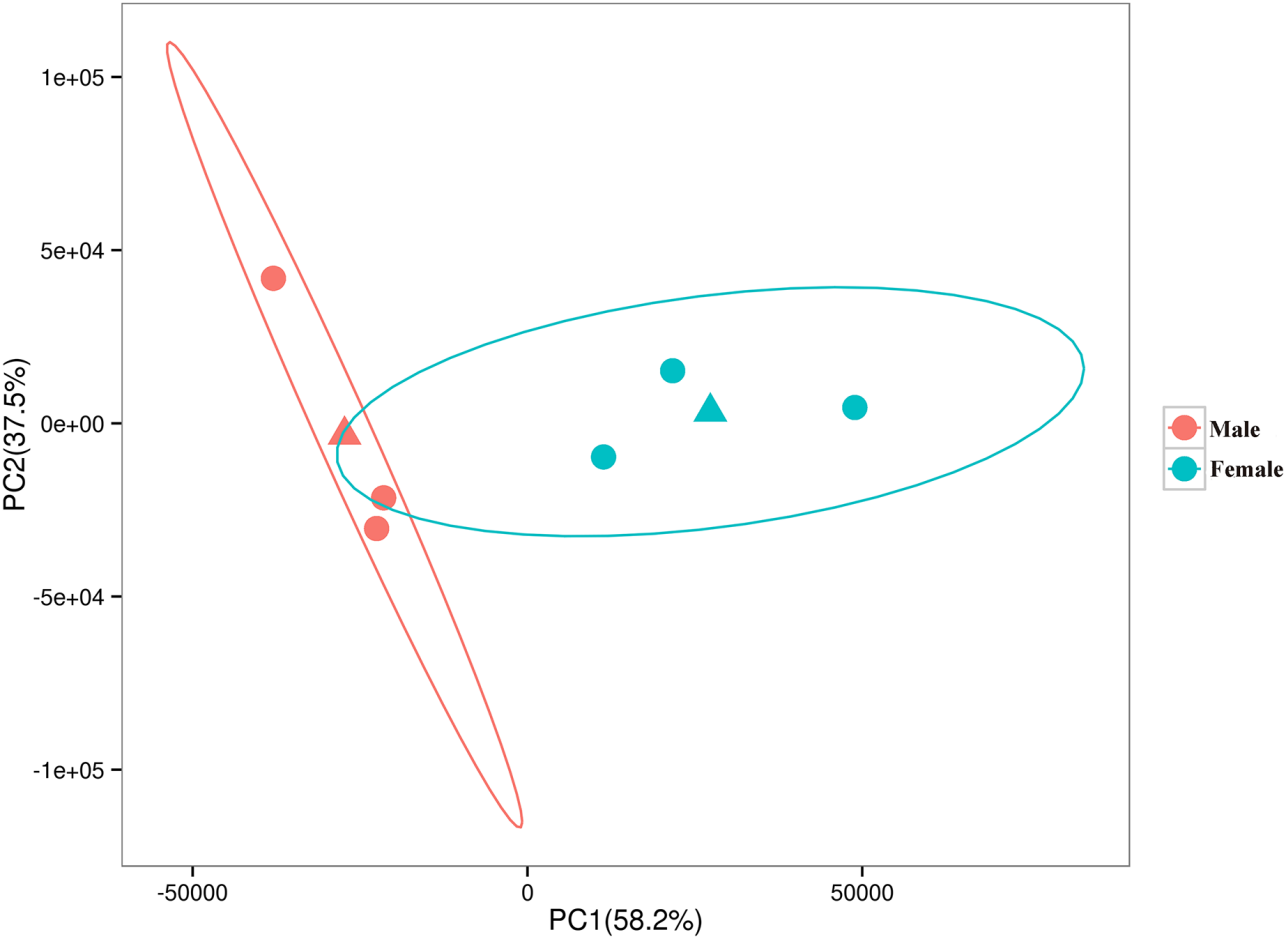
PCA plot of the six samples. The horizontal and vertical coordinates indicate the first and second principal components respectively, and the contribution of each principal component is shown in parentheses. The two triangles are the mean coordinates (centroids) of the male and female samples respectively.

**Figure 3:**
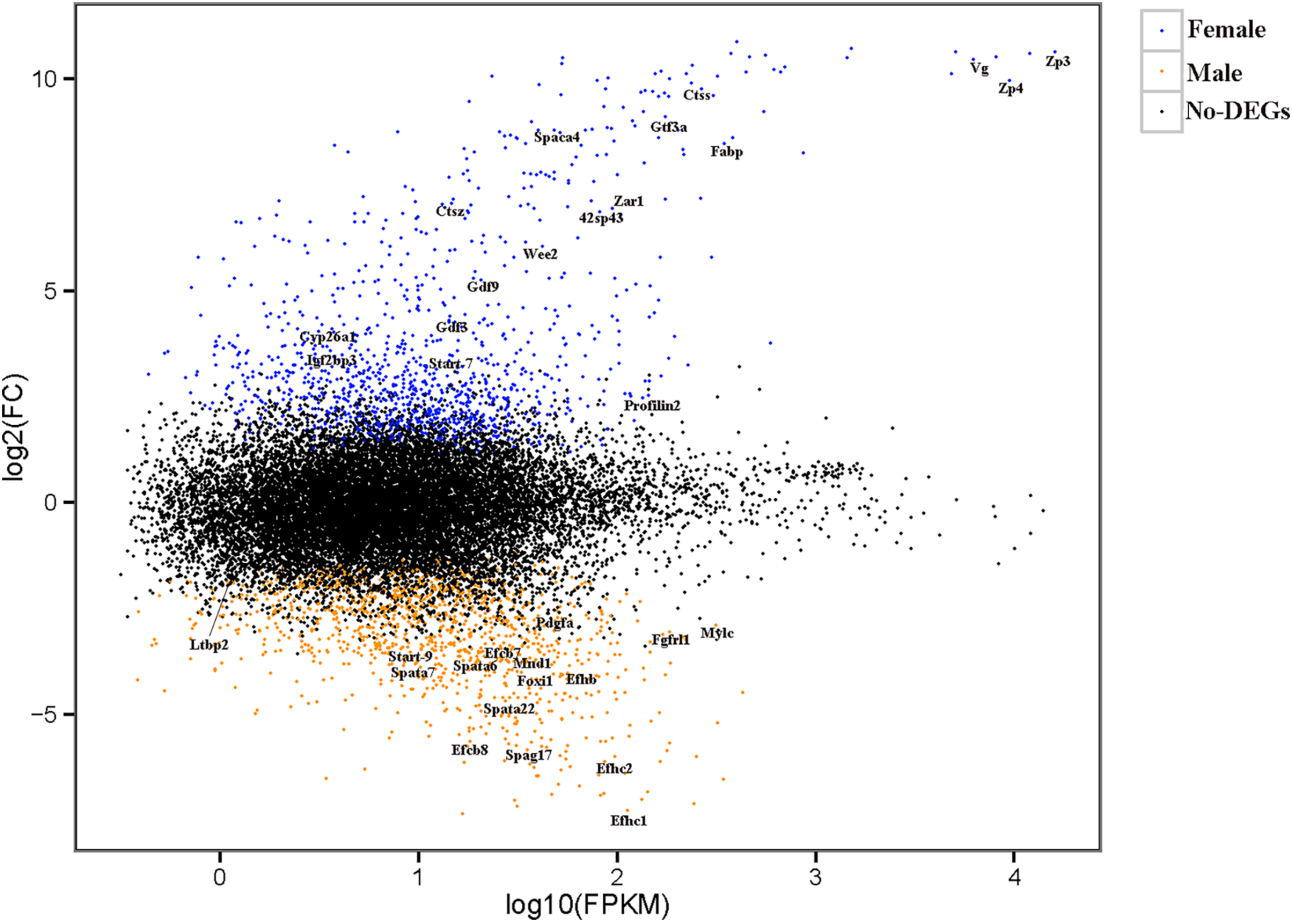
MA plot of DEGs. Each dot represents a gene. The blue and orange dots in the graph represent genes with significant expression differences. The blue dots are female-biased DEGs, the orange dots are male-biased DEGs and the black dots represent genes with no significant expression differences. The names of sex- and growth-related genes are showed in the figure.

The relationships among the six samples are shown in Fig. 2. Unigenes with Q-values ≤ 0.01 and |log_2_ fold change| ≥ 1 were identified as DEGs between female *D. macrosoma* and male *D. macrosoma* using DESeq2. After filtration, a total of 2,345 DEGs (Table S4) were obtained between male and female *D. macrosoma*, 1,150 of which were female-biased DEGs, while 1,195 unigenes were male-biased DEGs (Fig. 3).

#### DEG annotation

**Figure 4:**
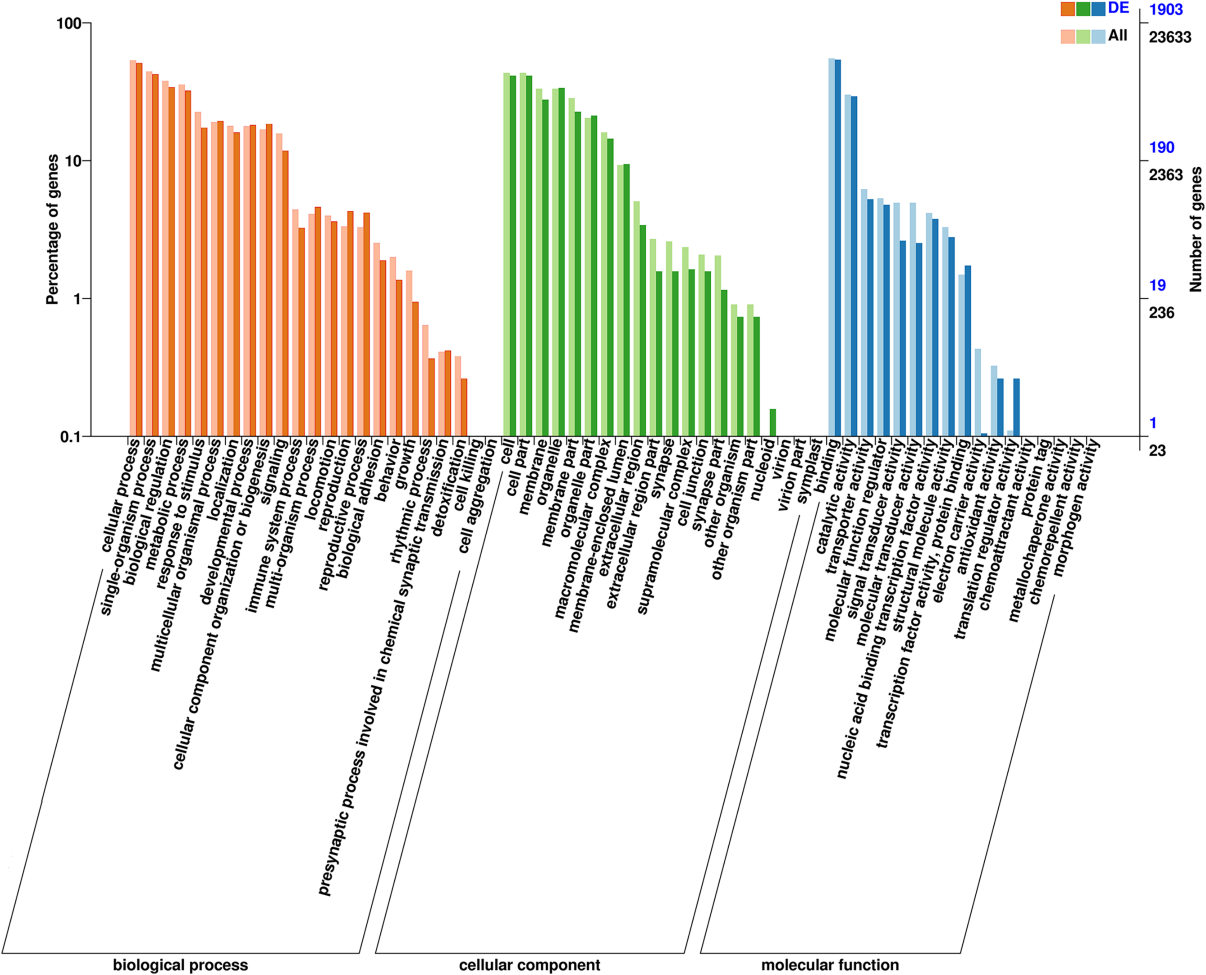
Clusters of GO categorization of the DEGs. The horizontal axis is the GO classification, the left side of the vertical axis is the percentage of the number of genes and the right side of the vertical axis is the number of genes. This figure shows the GO secondary function enrichment of the DEGs and all genes.

To explore possible differences in gene function between female and male *D. macrosoma*, GO and KEGG enrichment analyses were performed on the 2,345 DEGs identified between male and female *D. macrosoma* (Table S5). Visualization of the GO enrichment results (Fig. 4) showed that 1,903 unigenes were assigned to 60 GO terms (Table S6), and female and male *D. macrosoma* clearly differed in their biological processes. The top 10 enriched GO terms are listed in Table S7.

**Figure 5:**
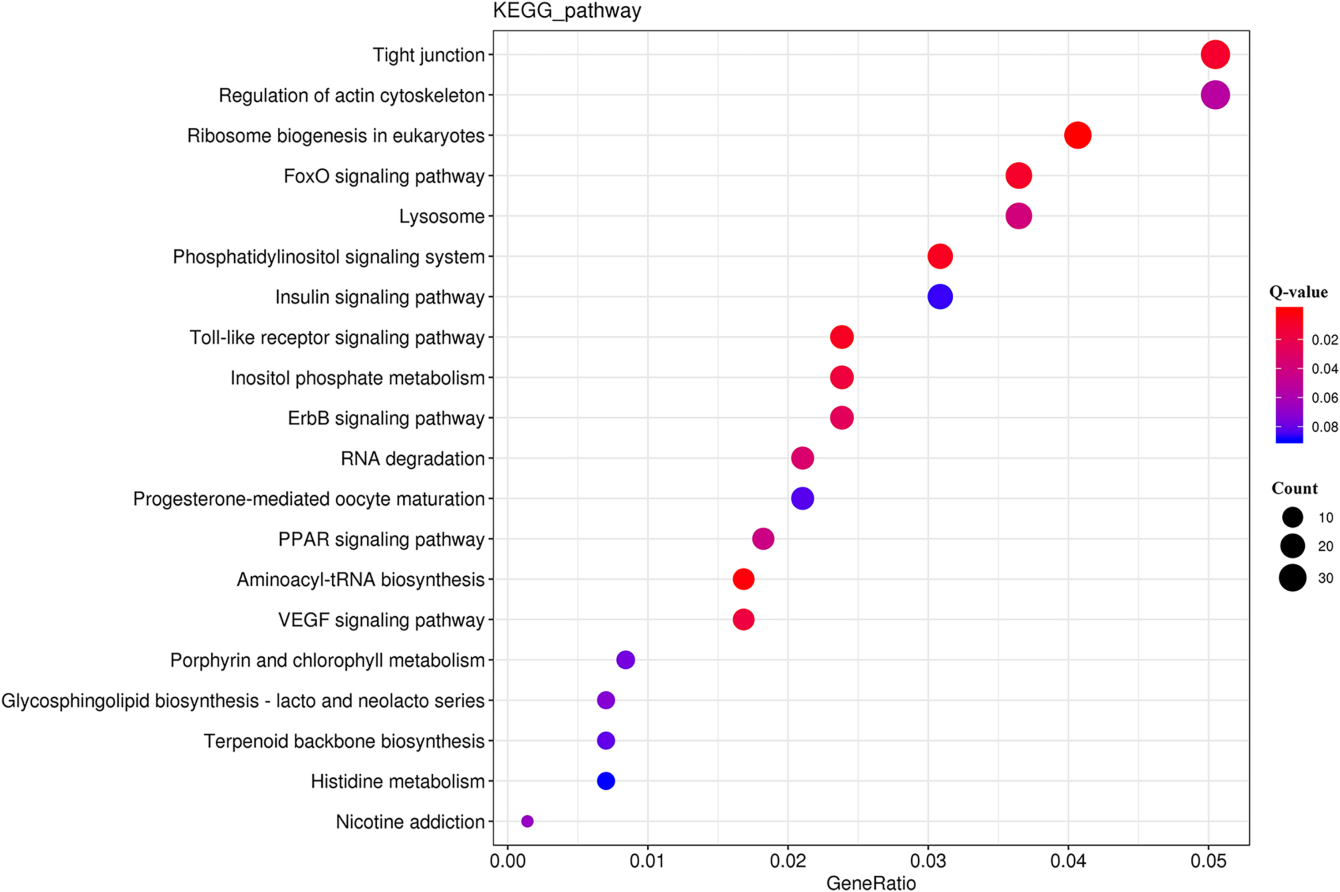
KEGG pathway enrichment analysis of the DEGs.

The KEGG analysis (Fig. S3) revealed that the DEGs were significantly enriched for 225 KEGG pathways (Table S8). The top 20 significantly enriched metabolic pathways (Fig. 5) included “tight junction” (ko04530), “regulation of actin cytoskeleton” (ko04810), and “ribosome biogenesis in eukaryotes” (ko03008).

According to the sequence annotation results, many DEGs were sex-controlled and gonad-development-associated genes, including the zona pellucida sperm-binding protein (*Zps*), sperm acrosome membrane-associated (*Spacas*), vitellogenin (*Vg*), cytochrome p450 enzyme (*Cyps*), stAR-related lipid transfer protein (*Start*), and sperm-associated antigen 17 (*Spag17*), as well as other potential candidate protein-encoding genes (Table 1). Growth, a complex trait, is jointly controlled by many genes. In this study, we identified multiple genes involved in growth regulation, such as genes related to fibroblast growth factor receptors, which control growth at the muscle tissue level and others (Table 2).

**Table 1:** Sex-related genes in *D. macrosoma*.

Gene	Annotation	Log2 (♀/♂)	Qvalue
*42sp43*	P43 5S RNA-binding protein-like isoform X1	6.85	1.40E−15
*Ctss*	Cathepsin S-like	9.9	1.48E−30
*Ctsz*	Cathepsin Z	5.14	1.09E−04
*Cyp26a1*	Cytochrome P450 26A1	4.11	3.84E−07
*Foxi1*	Forkhead box protein I1	−4.17	0
*Gtf3a*	Transcription factor IIIA	9.22	5.35E−32
*Mnd1*	Meiotic nuclear division protein 1 homolog isoform X1	−3.42	9.22E−03
*Profilin2*	Profilin-2-like isoform X1	1.94	0
*Spaca4*	Sperm acrosome membrane-associated protein 4-like isoform X1	9.62	2.16E−29
*Spag17*	Sperm-associated antigen 17	−5.74	4.39E−06
*Spata22*	Spermatogenesis-associated protein 22 isoform X2	−4.6	5.04E−04
*Spata6*	Spermatogenesis-associated protein 6-like isoform X2	−3.63	2.64E−03
*Spata7*	Spermatogenesis-associated protein 7	−3.75	3.53E−04
*Start-7*	StAR-related lipid transfer protein 7	1.55	0
*Start-9*	StAR-related lipid transfer protein 9	−3.38	7.03E−05
*Vg*	Vitellogenin-like	10.47	1.63E−65
*Wee2*	Wee1-like protein kinase 2	6.05	1.33E−12
*Zar1*	Zygote arrest protein 1	6.94	0
*Zp3*	Zona pellucida sperm-binding protein 3-like	8.85	1.32E−07
*Zp4*	Zona pellucida sperm-binding protein 4-like	9.34	1.82E−11

**Table 2:** Growth-related genes in *D. macrosoma*.

Gene	Annotation	Log2 (♀/♂)	Qvalue
*Efcb7*	EF-hand calcium-binding domain-containing protein 7	−3.62	0
*Efcb8*	EF-hand calcium-binding domain-containing protein 8	−5.75	1.49E−07
*Efhb*	EF-hand domain-containing family member B isoform X1	−4.05	9.56E−05
*Efhc1*	EF-hand domain-containing protein 1	−7.27	1.43E−10
*Efhc2*	EF-hand domain-containing family member C2	−6.44	2.75E−08
*Fabp*	Fatty acid-binding protein	8.47	9.34E−35
*Fgfrl1*	Fibroblast growth factor receptor-like 1	−3.44	1.74E−03
*Gdf3*	Growth differentiation factor 3	4.66	6.18E−06
*Gdf9*	Growth differentiation factor 9-like	5.91	4.97E−09
*Igf2bp3*	Insulin-like growth factor 2 mRNA-binding protein 3 isoform X3	3.59	7.53E−06
*Ltbp2*	Latent-transforming growth factor β-binding protein 2-like	−1.91	4.01E−03
*Mylc*	Myosin light chain	−3.8	8.72E−03
*Pdgfa*	Platelet-derived growth factor subunit A-like isoform X2	−2.86	7.63E−08

### SSR analysis

To explore genetic diversity, the SSRs of *D. macrosoma* were identified using MISA software. A total of 19,573 SSRs were located in 11,433 unigenes, 8,140 of which contained multiple SSRs. The frequency with which different SSRs appeared varied (Table S9), and mononucleotide SSRs had the highest frequency, accounting for 51.79% of total SSRs, followed by trinucleotide SSRs (22.63%). In addition, the relative SSR abundance varied greatly. For example, A/T was the most common mononucleotide SSR, while AC/GT had the highest frequency among dinucleotide SSRs.

## Discussion

### Transcriptome sequencing analysis of *D. macrosoma*

To identify the functions of genes expressed in the *D. macrosoma* and the biological processes in which they were involved, cDNA libraries were built from sexually mature female and male *D. macrosoma*. RNA-seq was performed on an Illumina platform, which yielded 42.61 Gb of clean data. N50 is an important parameter used to evaluate RNA-seq assembly quality. In this study, the N50 length of the transcriptome from *D. macrosoma* was 2,266 bp, and the BUSCO analysis showed that 92.9% of transcriptomes were complete. We concluded that the transcriptome of *D. macrosoma* was sufficiently complete and well assembled; therefore, it may be valuable for gene function analyses.

### Functional annotation

Nearly half of *D. macrosoma* unigenes were not annotated to any sequences in the reference databases. The low annotation ratio seems unsurprising in non-model organisms without published genomes (Jung et al., 2011b; Robledo et al., 2014; Ma et al., 2016). Previous studies on transcriptome analyses indicate that unannotated sequences mainly represent transcripts spanning only untranslated mRNA regions, chimeric sequences derived from assembly errors (Wang et al., 2004), and sequences containing non-conserved protein regions (Mittapalli et al., 2010). Some may also be components of novel genes specific to this species, which are likely to be matched to certain genome sequences in the near future (Ma et al., 2016). A comparison against the Nr database showed that 29% (7,552 of 26,514 unigenes) of the comparable sequences were homologous to *S. partitus* sequences, and 24% (6,312 of 26,514 unigenes) were homologous to *L. crocea* sequences (Fig. S2), indicating that *D. macrosoma* was closely related to *S. partitus* and *L. crocea*. In the *D. macrosoma* transcriptome, “cellular anatomical entity”, “cellular process”, and “binding” were the most enriched under the three major GO categories (Fig. 4). The results are similar to RNA-seq results for other aquatic animals, suggesting that the genes in these functional classes are conserved among species (Wang, Lou & Shui, 2019; Sun & Han, 2019). As indicated by the KEGG annotation results, the unigenes in the *D. macrosoma* transcriptome mainly participated in the “MAPK signaling pathway” (569), “endocytosis” (544), and “calcium signaling pathway” (502), indicating that cellular physiological pathways may largely rely on the regulation of functional gene expression, since functional genes are important specific mediators and effectors of the cell physiological activities of living organisms (DeMeester, Buchman & Cobb, 2001).

### DEGs in female and male *D. macrosoma*

In this study, a total of 2,345 DEGs were detected in the transcriptomes of female and male *D. macrosoma*, 1,150 of which were highly expressed in female *D. macrosoma*, while 1,195 were highly expressed in male *D. macrosoma*. Compared with males, female *D. macrosoma* have a significantly faster growth rate. These DEGs might be associated with sex determination or correlated with growth. Our findings lay a foundation for further explorations of the molecular mechanisms underlying biological processes in *D. macrosoma*.

In this study, many growth- and sex-related genes were detected in the *D. macrosoma* transcriptome data. A large number of studies have shown that genes encoding growth axis components, including insulin-like growth factors (*Igf*), somatostatin, and their carrier proteins and receptors (De-Santis & Jerry, 2007), played crucial roles in regulating the formation of skeletal muscles in finfish.

The GO enrichment analysis of the DEGs revealed clearly different biological processes between female and male *D. macrosoma*, including growth, rhythmic processes, and immune defense. The GO enrichment analysis of unigenes indicated that the significantly enriched GO terms were mainly correlated with cilia and axonemes, including cilium assembly, cilium tissue, axoneme assembly, and the axonemal dynein complex. Cilia, which extend from the top of the matrix (from the centriole) and extrude from microtubule-based hairlike organelles on the cell surface, function in motility and signal transduction and can regulate animal reproduction, development, and perception (Wei et al., 2016). The axonemal dynein complex not only constitutes the dynein arms of peripheral doublets in cilia or flagella but also powers the mutual sliding of peripheral doublets (Zhao et al., 2020). These GO terms were significantly more highly expressed in males than in females, indicating that cilia and axonemes may be more critical in *D. macrosoma* males than in females.

The KEGG enrichment analysis revealed that some sex-related pathways, including “MAPK signaling pathway”, “neuroactive ligand-receptor interaction”, “GnRH signaling pathway”, “TGF-beta signaling pathway”, and “p53 signaling pathway”. The MAPK signaling pathway is present in all eukaryotes. The MAPK signaling pathway is mainly involved in the regulation of gonadotropin subunit gene expression and the movement of primary spermatocytes across the blood-testis barrier (Di et al., 2002). It has a wide range of cellular roles in growth, differentiation, and stress responses (Jung et al., 2011a). In the study, the MAPK signaling pathway was significantly enriched in male *D. macrosoma* and was also enriched in female *D. macrosoma*, although without statistical significance, which might indicate that the MAPK signaling pathway plays different roles in the gonadal development of *D. macrosoma*. The neuroactive ligand-receptor interaction pathway is related to lactation performance in mice. In addition, genes related to this pathway have been found to be upregulated (Wei et al., 2013). Previous studies have shown that the neuroactive ligand-receptor interaction pathway plays an important role in teleost reproduction and gonadal development (Du et al., 2017; Li et al., 2017). Therefore, the neuroactive ligand-receptor interaction pathway may have an effect on gonadal development and sexual maturity in *D. macrosoma*.

Regarding calcium ion regulation, many biological reactions are triggered or regulated through subtle changes in the intracellular calcium ion concentration. The precise sensing of intracellular calcium ion concentrations within different ranges by EF-hand proteins (as intracellular sensors) is the key to this regulatory process. The regulatory function of signaling *via* EF-hand proteins is mainly ascribed to their calcium ion-induced conformational changes, which consequently expose many hydrophobic target protein binding sites. Moreover, the functions of buffer proteins, which regulate calcium ion concentration, are affected by the selectivity of calcium ions, the dynamics of binding calcium ions, *etc*. Calmodulin (CaM) and related protein-coding (EF-hand superfamily) genes participate in several cellular physiological processes, including development, growth, and cell differentiation, and play significant roles in the nervous system, immune system, reproductive system, motor system, *etc*. (Qi et al., 2015). Past studies have shown that the EF-hand superfamily was significantly expressed in *C. myriaster* (Chen, 2019). EF-hand proteins are involved in regulating almost all cellular functions. For instance, CaM-dependent protein kinases have important effects on the regulation of the immune and nervous systems and on sperm formation (Zhu, 2008). Hence, the EF-hand protein family may directly or indirectly regulate cell growth in *D. macrosoma*. In this study, five EF-hand proteins (*i.e*., EF-hand domain-containing protein 1, EF-hand domain-containing family member C2, EF-hand calcium-binding domain-containing protein 7, EF-hand calcium-binding domain-containing protein 8, and EF-hand domain-containing family member B isoform X1) were screened from the *D. macrosoma* transcriptome. They all showed higher expression in males than in females, indicating that CaM and related protein-coding genes may have vital functions in male *D. macrosoma* growth and reproduction.

Fatty acid-binding proteins (FABPs) are a group of proteins that coordinate lipid trafficking and signaling in cells. The proteins play an important role in facilitating cellular uptake and transfer of fatty acids (FAs), targeting FAs to specific metabolic pathways, and participating in the regulation of gene expression and cell growth (Haunerland & Spener, 2004). As shown in Table 2, *Fabp* showed significantly higher expression in female *D. macrosoma* than in males, indicating that the *Fabp* gene may play critical roles in sex differentiation in *D. macrosoma*. Thus far, information on the progress of the fish *Fabps* gene is limited to the expression patterns in *Atlantic salmon* (Torstensen et al., 2009) and promoter function in zebrafish (Her, Chiang & Wu, 2004). Therefore, a broad area of interest remains regarding exploration of the functions of the FABP family in teleost fish (Ma et al., 2016).

Insulin-like growth factors (IGFs) and relevant carrier proteins are some of the most important components of the somatotropic axis. They are widely recognized to play a significant role in regulating the formation of skeletal muscles in finfish (De-Santis & Jerry, 2007). In this study, compared to the male *D. macrosoma*, female *D. macrosoma* showed significantly higher *Igf2bp3* expression levels, suggesting that *Igf2bp3* may play an important role in the reproduction and growth of *D. macrosoma*.

The zona pellucida is the extracellular matrix around oocytes, protecting them and playing an important role in their fusion with sperm (Dumont & Brummett, 1985). The zona pellucida comprises four glycoproteins (ZP1-4), which bind to each other to form long filaments (Wassarman, 1988; Litscher & Wassarman, 2018). Zona pellucida proteins were first discovered in mammalian egg membranes. They were later discovered in the inner layer of fish chorions (Litscher & Wassarman, 2007). Studies have found that ZP3 was a primary female-specific reproductive molecule (Liu, Wang & Gong, 2006). Recently, a number of copies of the *Zp* genes have been identified in some teleost species, such as carp and medaka (Chang et al., 1997; Kanamori et al., 2003). *Zp3* and *Zp4* function as major sperm receptors and induce acrosome reaction (Litscher & Wassarman, 2007). In this study, the expression levels of *Zp3* and *Zp4* in females were higher than those in males, showing that these genes may play vital roles in ovarian development in fish and implicating their roles in *D. macrosoma* genesis and reproduction.

Vitellogenin (Vg), a protein found in mature females of nonmammary oviparous animals, functions as the precursor for vitellin in nearly all oviparous animals (Liu et al., 2016). Synthesized by hypothalamic-pituitary-gonadal (HPG) axis-activated estrogen receptors (ERs), Vg is a critical substance in yolk synthesis and an important reproductive protein in fishes. Vg also plays an important role in the reproduction and development of oviparous animals (Wu, 2018). In oviparous animals, Vg not only provides saccharides, fats, proteins, and other nutrients for the development of mature oocytes into embryos but also binds and transports metal ions (Zn^2+^, Fe^3+^, Cu^2+^, Mg^2+^ and Ca^2+^), carotenoids, thyroxines, retinols, and riboflavin into oocytes (Valle, 1993; Taborsky, 1980; Babin, 1992; Azuma, Irie & Seki, 1993; Mac, Nimpf & Schneider, 1994). The vast majority of studies suggest that although the *Vg* gene exists in larvae and males, it can be expressed only by adult females. Although Vg is a female-specific protein (including in fishes), estrogen-induced anomalies can result in its production in male fishes. Hence, Vg has been widely investigated as a sensitive biomarker for monitoring environmental estrogen-like substances (Wallace, 1985; Specker & Sullivan, 1994). Unuma et al. (1998) detected the expression of the *Vg* gene in the gonads of both female and male *Pseudocentrotus depressus*, and its expression gradually decreased with testis development. We inferred that Vg could be utilized as a nutrient substance for sperm development. Studies on *Halocynthia roretzi* (Akasaka, Harada & Sawada, 2010; Akasaka et al., 2013) have shown that the vWFD and CT structural domains in the C-terminus of the Vg protein may be positioned in the vitellin coat and bind to two enzymes in sperm (HrProacrosin and HrSpermosin), thereby participating in the fertilization process once sperm has entered eggs (Liu et al., 2016). In this study, *Vg* was significantly upregulated in females compared with males. Therefore, its expression in the testes may serve as a nutrient substance for spermatogenesis or may assist in fertilization, indicating that *Vg* may be especially important in the reproduction and development of both female and male *D. macrosoma*.

### SSR markers

SSRs, a significant class of molecular markers, are important resources in population genetics studies. In this study, 19,573 SSRs were acquired from 11,433 unigenes. Among the identified SSRs, mononucleotide SSRs were the most abundant, while fewer SSRs had more repetitive nucleotides (Table S9), which was consistent with the results of the SSR analysis in *Oplegnathus punctatus* (Du et al., 2017), *Charybdis feriata* (Zhang et al., 2017), and *Sillago sihama* (Tian et al., 2019). The *D. macrosoma* SSRs acquired in this study provide data that can be used to explore genetic diversity and aid in constructing genetic linkage maps and developing genetic resources for this species.

## Conclusions

This study is the first to generate transcriptome data from female and male *D. macrosoma*. A total of 58,475 unigenes were obtained through assembly, and then 28,174 unigenes were functionally annotated, thereby enriching the functional genetic resources for this species. Many candidate genes related to growth and sex differentiation were detected by comparing transcriptomes from female and male *D. macrosoma*. A total of 19,573 SSR sites were obtained from 11,433 *D. macrosoma* unigenes, which yielded data to aid in studying the genetic diversity of this species. There are extremely limited genome assembly data for Carangidae fishes in public databases, and no genome assembly data exist for *Decapterus*. This study supplies valuable information about the growth- and sex-related genes of the Osteichthyes, fills the gap in public genome assembly data for *Decapterus*, lays a foundation for further study of Carangidae fishes, and facilitates further investigations of fish transcriptomes.

## Supplemental Information

10.7717/peerj.14342/supp-1 Supplemental Information 1 Author checklist - full.

10.7717/peerj.14342/supp-2 Supplemental Information 2 Supplementary figures and tables.

10.7717/peerj.14342/supp-3 Supplemental Information 3 Supplementary tables.

## Additional Information and Declarations

### Data Availability

The following information was supplied regarding data availability: The data is available at NCBI Sequence Read Archive (SRA): PRJNA825736, SRR18748694–SRR18748699.
