# Supplementary material for: Comparative transcriptome sequencing analysis of female and male Decapterus macrosoma
Source: PeerJ. 2022 Nov 8;10:e14342. doi: 10.7717/peerj.14342 (PMC9651050; doi:10.7717/peerj.14342)
Supplement: Supplemental Information 2 [file peerj-10-14342-s002.docx]

**Table S1 Summary statistics of clean transcriptome sequencing data from each sample.**

| Sample | Read count | Base count | GC content | Clean reads Q30 |
| --- | --- | --- | --- | --- |
| T01  T02  T03  T04  T05  T06 | 22,382,287  22,020,004  24,747,208  25,547,319  26,513,859  21,306,701 | 6,683,773,680  6,577,048,480  7,399,616,482  7,641,161,830  7,934,181,004  6,372,930,036 | 51.71%  51.32%  51.23%  50.94%  51.41%  50.98% | 93.78%  93.43%  93.90%  93.77%  93.82%  93.73% |

T01, T02, and T03 represent male *D. macrosoma* samples; T04, T05, and T06 represent female *D. macrosoma* samples. Read count: the number of the pair-end reads in the clean data. Base count: the base number in the clean data. GC content: the percentage of G and C bases in total bases.

**Table S2 Statistics for the assembled unigenes.**

| Sample | Mean Length | N50 | GC (%) | Mapped ratio |
| --- | --- | --- | --- | --- |
| T01  T02  T03  T04  T05  T06 | 850  868  839  890  877  874 | 1576  1734  1638  1719  1786  1636 | 47.58  47.88  47.72  48.27  48.47  48.24 | 84.05%  78.93%  80.37%  83.66%  83.42%  84.28% |
| All Unigenes | 1284 | 2,266 | 47.12 | 100% |

T01, T02, and T03 represent male *D. macrosoma* samples; T04, T05, and T06 represent female *D. macrosoma* samples. Mean Length indicates the average length of unigenes. N50 indicates the length of N50. GC (%): the percentage of G and C bases. Mapped ratio indicates the proportion of mapped unigenes per sample in all unigenes.

**Table S3 Summary of unigenes annotations.**

| Database | Number of unigenes | Percentage (%) |
| --- | --- | --- |
| GO  KEGG  KOG  Pfam  Swiss-Prot  TrEMBL  eggNOG  Nr  At least one database  Total unigenes | 23,633  22,890  17,022  20,264  13,871  27,502  23,515  27,402  28,174  58,475 | 40.42%  39.14%  29.11%  34.65%  23.72%  47.03%  40.21%  46.86%  48.18%  100.00% |

**Table S7 Significant enrichment analysis of GO terms of unigenes that are differentially expressed between female and male *D. macrosoma*.**

| GO ID | GO term | Mapped genes | Genome background | Q-value |
| --- | --- | --- | --- | --- |
| GO:0060271 | Cilium assembly | 57 | 252 | 1.23e-09 |
| GO:0035082 | Axoneme assembly | 21 | 44 | 5.57e-09 |
| GO:0044782 | Cilium organization | 46 | 193 | 2.51e-07 |
| GO:0006364 | rRNA processing | 35 | 133 | 3.99e-07 |
| GO:0005929 | Cilium | 71 | 276 | 1.00e-12 |
| GO:0005858 | Axonemal dynein complex | 12 | 16 | 1.33e-08 |
| GO:0005930 | Axoneme | 28 | 69 | 1.33e-08 |
| GO:0097014 | Ciliary plasm | 28 | 69 | 1.56e-08 |
| GO:0044441 | Obsolete ciliary part | 50 | 195 | 1.89e-08 |
| GO:0035804 | Structural constituent of egg coat | 6 | 6 | 0.000201 |

**Table S9 SSRs from the *D. macrosoma* transcriptome.**

| Type | Count |
| --- | --- |
| Total sequences | 58,475 |
| Total SSR sites | 19,573 |
| Sequences containing SSR sites | 11,433 |
| Sequences containing >1 SSR site | 8,140 |
| Hybrid SSR-c | 1,364 |
| Hybrid SSR-c* | 22 |
| Mononucleotide SSRs | 10,137 |
| Dinucleotide SSRs | 3,367 |
| Trinucleotide SSRs | 4,430 |
| Tetranucleotide SSRs | 231 |
| Pentanucleotide SSRs | 12 |
| Hexanucleotide SSRs | 10 |

c: compound repeat SSRs; c*: compound SSRs with overlap.


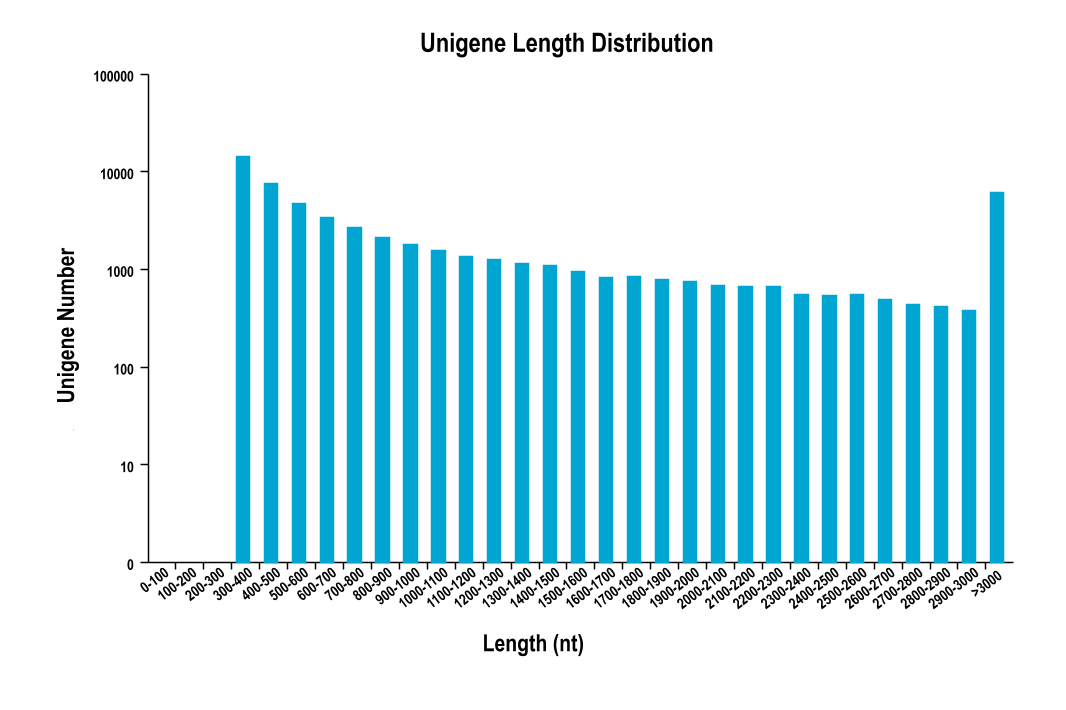


**Figure S1** **Length distribution of unigenes** **in the *D. macrosoma* transcriptome.**


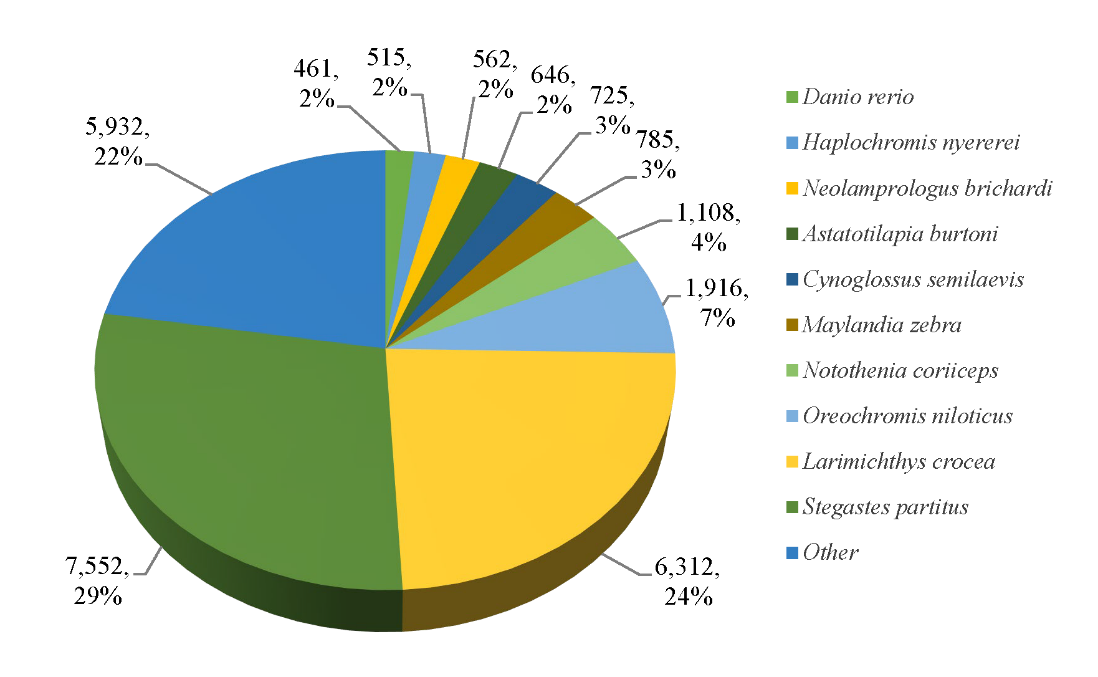


**Figure S2 Distribution of *D. macrosoma* unigene homology relative to other species.**

The *D. macrosoma* unigenes are highly similar to gene sequences from *Stegastes partitus* (7,552), *Larimichthys crocea* (6,312), *Oreochromis niloticus* (1,916), *Notothenia coriiceps* (1,108), *Maylandia zebra* (785), *Cynoglossus semilaevis* (725), *Astatotilapia burtoni* (646), *Neolamprologus brichardi* (562), *Haplochromis nyererei* (515), *Danio rerio* (461) and other species (5932).


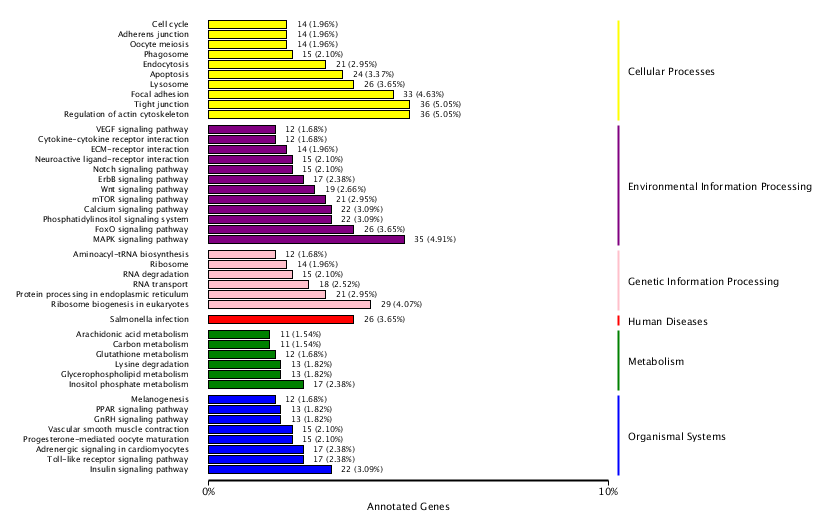


**Figure S3 KEGG classification of the DEGs.**

The horizontal axis is the ratio of DEGs annotated to the pathway over all DEGs. The values beside the bars represents the number of DEGs involved in this pathway. The color of bars stands for classification of the pathways. The vertical axis is name of KEGG pathways.
